# Isotopic Analysis of Sporocarp Protein and Structural Material Improves Resolution of Fungal Carbon Sources

**DOI:** 10.3389/fmicb.2016.01994

**Published:** 2016-12-26

**Authors:** Janet Chen, Kirsten S. Hofmockel, Erik A. Hobbie

**Affiliations:** ^1^Earth, Oceans and Space, Earth Systems Research Center, University of New HampshireDurham, NH, USA; ^2^Soil and Water Management and Crop Nutrition Laboratory, FAO/IAEA Agriculture and Biotechnology LaboratoriesSeibersdorf, Austria; ^3^Department of Ecology, Evolution and Organismal Biology, Iowa State UniversityAmes, IA, USA; ^4^Environmental Molecular Sciences Laboratory and Biological Sciences Division, Pacific Northwest National LaboratoryRichland, WA, USA

**Keywords:** organic nitrogen use, ^13^C, ^15^N, ectomycorrhizal fungi, saprotrophic fungi, carbon uptake

## Abstract

Fungal acquisition of resources is difficult to assess in the field. To determine whether fungi received carbon from recent plant photosynthate, litter or soil-derived organic (C:N bonded) nitrogen, we examined differences in δ^13^C among bulk tissue, structural carbon, and protein extracts of sporocarps of three fungal types: saprotrophic fungi, fungi with hydrophobic ectomycorrhizae, or fungi with hydrophilic ectomycorrhizae. Sporocarps were collected from experimental plots of the Duke Free-air CO_2_ enrichment experiment during and after CO_2_ enrichment. The differential ^13^C labeling of ecosystem pools in CO_2_ enrichment experiments was tracked into fungi and provided novel insights into organic nitrogen use. Specifically, sporocarp δ^13^C as well as δ^15^N of protein and structural material indicated that fungi with hydrophobic ectomycorrhizae used soil-derived organic nitrogen sources for protein carbon, fungi with hydrophilic ectomycorrhizae used recent plant photosynthates for protein carbon and both fungal groups used photosynthates for structural carbon. Saprotrophic fungi depended on litter produced during fumigation for both protein and structural material.

## Introduction

Despite the diverse roles that fungi play in carbon cycling in forest ecosystems, little is known about how different fungi partition carbon resources internally. Instead, fungi in forest ecosystems are commonly classified by the substrates they use. Saprotrophic fungi dominate the litter layer (Oi horizon) and decaying material such as litter, cones and wood (Rayner and Boddy, 1988). Ectomycorrhizal fungi are primarily active in the organic horizons below the litter layer, decompose relatively older organic matter for resources, and can transfer acquired nutrients to plants in exchange for plant photoassimilates (Smith and Read, 1997; Lindahl et al., 2007). Ectomycorrhizal fungi can be classified into those with hydrophobic ectomycorrhizae or hydrophilic ectomycorrhizae (Unestam and Sun, 1995; Agerer, 2006) that purportedly can use insoluble or soluble substrates, respectively (Lilleskov et al., 2011; Hobbie et al., 2013b). Specifically, fungi with hydrophobic ectomycorrhizae consist of hyphae with medium to long distance exploration types that can use insoluble as well as soluble substrates and fungi with hydrophilic ectomycorrhizae consist of hyphae with contact and shorter distance exploration types that rely on soluble substrates. While these generalized classifications are helpful, quantifying which carbon sources are accessed by specific taxa requires new approaches.

In laboratory studies, fungi can be supplied with specific carbon substrates to determine which ones are preferentially consumed (Tinker et al., 1990; Colpaert et al., 1996; Hobbie et al., 2004). These studies provide insight into fungal uptake capabilities, but they may not represent fungal resource acquisition strategies in the field. In the field, labeling with isotope tracers can track fungal sources of carbon (Ho and Trappe, 1973; Högberg et al., 2010; Hobbie et al., 2014). Such labeling has included elevated atmospheric CO_2_ experiments, in which ^13^C-depleted CO_2_ is added to raise CO_2_ concentrations in free air CO_2_ enrichment (FACE) studies. Carbon isotope signatures in bulk sporocarp material of ectomycorrhizal and saprotrophic fungi confirmed that both types of fungi rely on carbon recently assimilated by plants and suggested that some ectomycorrhizal fungi also incorporated carbon from soil-derived organic nitrogen sources that predated the ^13^C labeling (Hobbie et al., 2014). Additional studies have measured isotopic composition of sporocarps at natural abundance levels to determine fungal sources of carbon, with the ^13^C:^12^C ratio (δ^13^C) generally lower for ectomycorrhizal fungi than for saprotrophic fungi (Högberg et al., 1999; Kohzu et al., 1999; Hobbie et al., 2001). The ^15^N:^14^N ratio (δ^15^N) of fungi has also been used to determine fungal sources of organic (carbon-bonded) nitrogen, since deeper and older forms of organic nitrogen are ^15^N-enriched relative to shallower and younger organic nitrogen (Billings and Richter, 2006; Hobbie and Ouimette, 2009). This difference in δ^15^N across soil profiles is largely due to inputs of low δ^15^N litter higher in the soil profile and decomposition that increases δ^15^N values deeper down in the soil profile (Nadelhoffer and Fry, 1988), as well as due to fractionation of N during ecotmycorrhizal transfer of N to plants (Hobbie and Colpaert, 2003). Studies to date have focused on bulk sporocarps and have given significant but limited insight into fungal sources of carbon.

Isotope analysis of different fungal compounds (e.g., protein, chitin, and lipids) can provide new information about how carbon is allocated, but this has only begun to be studied. Hobbie et al. (2012) suggested that fungal protein was enriched in ^13^C and ^15^N relative to fungal carbohydrates, chitin, and lipids based on bulk isotopic patterns in protein-rich caps relative to protein-poor stipes of sporocarps. Radiocarbon ^14^C studies on protein and structural material of fungi with hydrophobic and hydrophilic ectomycorrhizae indicate that structural carbon is derived from recent carbon sources, such as photosynthate, while protein carbon can be derived from new photosynthate and from either recent organic nitrogen in the younger Oi horizon or from older organic nitrogen sources in the Oea horizon, depending on the fungal functional group (Hobbie et al., 2013a). Glycoproteins and polysaccharides are the major structural compounds in fungal cell walls that facilitate formation and structure of fungi (Bowman and Free, 2006). Chitin is also an integral component of the fungal cell wall (Bago et al., 1996; Bowman and Free, 2006). Additional proteins are involved in metabolism, protection, growth and maintenance (Persson et al., 1991; Niessing, 2000; Saier, 2000; González-Chávez et al., 2004; Seidl, 2008). Because most carbon is allocated to structural and protein material in fungi (Kalač, 2009), and because of the foundation of these previous studies, structural and protein extracts of sporocarps are good candidates for characterizing carbon acquisition and allocation.

**FIGURE 1:**
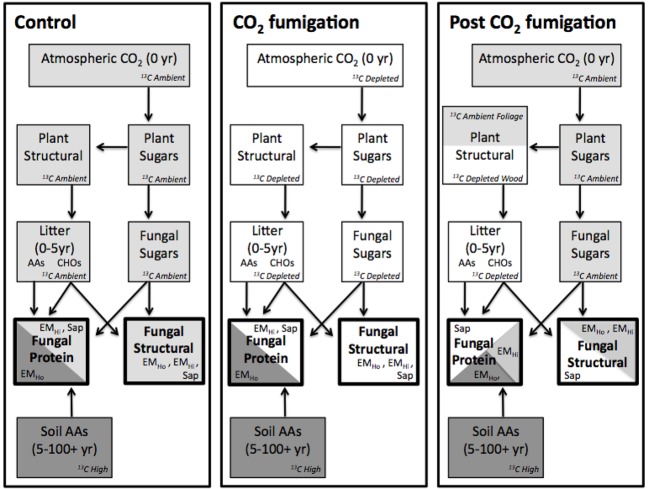
**Schematic of carbon fluxes in the plant-fungal-soil system, showing how uptake of recently assimilated carbon and old soil organic nitrogen can influence the δ^13^C of saprotrophic (Sap) fungi of fungi with hydrophilic ectomycorrhizae (EM_Hi_) and hydrophobic (EM_Ho_) ectomycorrhizae fungi when carbon sources differ in δ^13^C.** Scenarios are represented for ambient, CO_2_-fumigated and previously CO_2_-fumigated FACE plots. AAs = amino acids. ^13^C high to low from dark gray > light gray > white.

Here we determined sources of ectomycorrhizal and saprotrophic carbon using sporocarps and soil collected from the Duke FACE experiment. CO_2_-fumigated plots in a *Pinus taeda* plantation were fumigated with ^13^C-depleted CO_2_ from 1997 to 2010 (Hendrey et al., 1999). Differences in δ^13^C of bulk tissue, structural carbon, and protein extracts from ambient and FACE treatment plots during and after CO_2_ enrichment were used to determine whether fungi obtained carbon from recent plant photosynthate, plant litter, or soil-derived organic nitrogen sources. To further clarify whether carbon from soil organic matter was acquired from the younger or older organic soil horizons, patterns of soil and fungal δ^15^N were also investigated. We predicted that bulk saprotrophic sporocarps collected post-CO_2_ fumigation from wood decay and litter decay groups would be lower in δ^13^C than ectomycorrhizal fungi due to the acquisition of litter-derived carbon produced during CO_2_ fumigation. Furthermore, δ^13^C and δ^15^N patterns in protein and structural carbon of sporocarps tested whether fungi with hydrophobic ectomycorrhizae use soil-derived organic nitrogen sources for protein carbon, fungi with hydrophilic ectomycorrhizae use recent plant photosynthates for protein carbon, and whether both fungal groups depend on plant photosynthates for structural carbon as opposed to saprotrophic fungi relying on recent litter and wood (**Figure 1**). Specifically, we predicted that protein of fungi with hydrophobic ectomycorrhizae would be lower in δ^13^C than structural material after CO_2_ fumigation shutoff, reflecting older, deeper organic N sources, while protein and structural material of fungi with hydrophilic ectomycorrhizae and saprotrophic fungi would be similar, reflecting recent assimilates and surficial litter created before CO_2_ shutoff. We also predicted that the δ^15^N of fungi with hydrophobic ectomycorrhizae would be higher than fungi with hydrophilic ectomycorrhizae and saprotrophic fungi, reflecting the differing sources of organic N in the soil profile.

## Materials and Methods

### Field Site

Our work was conducted within the Duke Forest FACE experiment in North Carolina, USA (35°58′41″N, 79°05′39″ W, 163 m elevation). The forest is dominated by loblolly pine (*Pinus taeda*) that were planted in 1983 as 3-year-old seedlings with 2.4 m^2^ spacing. Deciduous tree species including *Liquidambar styraciflua*, *Acer rubrum*, *Cercis Canadensis*, and *Cornus florida* dominate the understory. Three control and three CO_2_-fumigated plots, 30 m in diameter, were fumigated with ambient air or CO_2_ (to 200 ppm above ambient levels), respectively. The CO_2_ used for fumigation was strongly depleted in ^13^C relative to atmospheric CO_2_, with a δ^13^C of -43.0 ± 0.6‰, thus lowering the δ^13^C of CO_2_ within the ring by 12‰, to -20‰. Fumigation began August 27, 1996 and was continuous until fumigation shutoff on November 1, 2010. A 98 atom% ^15^N tracer (75% as NH_4_Cl and 25% as KNO_3_ at a rate of 15 mg ^15^N m^-2^ in 0.25 l H_2_O) was applied by backpack sprayers to all of each plot in May 2003. This represented 3% of the inorganic N pool from 0 to 15 cm depth. Additionally, in 2005 each FACE plot was divided in half with a plastic sheet to a 70 cm depth. Half of the plot was fertilized with 56 kg ha^-1^ unlabeled NH_4_NO_3_ twice, in March and April 2005, and then in one annual application in March until CO_2_ fumigation shutoff in 2010. Although the added ^15^N label in our plots raised ^15^N levels in our sporocarps compared to other studies without a tracer addition and raised δ^15^N of the Oea soil horizon relative to mineral soils, we were still able to compare δ^15^N values between fungal types, CO_2_ treatments and protein and structural material extracts within our study. Additionally, fertilization did not significantly affect soil or fungal δ^15^N values. The soil is a fine sandy loam classified as being from the Enon Series (fine, mixed, active, thermic Ultic Hapludalfs). The pH is 5.75 and derived from mafic bedrock. There are well-developed soil horizons with mixed clay mineralogy.

### Soil and Sporocarp Sampling

Soils were collected in November 2010 and April 2013 using methods described in Hofmockel et al. (2011). Soils were separated by depth into organic Oi, Oea horizons, and mineral 0-15 cm and 15-30 cm horizons and then sieved to 2 mm, dried and homogenized. Sporocarps were collected in October 2000, August and December of 2001, January, February, September, and November of 2002, October 2004, October 2010 and September and October of 2012. On each sampling day, each ring was surveyed and all sporocarps were collected. Whole sporocarps were dried, identified, and then homogenized to a fine powder and stored at room temperature until further analysis. Among the fungal groups, fungi with hydrophilic ectomycorrhizae (as noted in previous fungal literature) consisted of sporocarps from the genera *Amanita, Chroogomphus, Hygrophorus, Inocybe, Laccaria, Lactarius*, and *Russula.* Fungi with hydrophobic ectomycorrhizae (also as noted in fungal literature) consisted of sporocarps from the genera *Cortinarius, Hydnellum, Sistotrema, Suillus, Tricholoma*, and *Tylopilus*. Saprotrophic wood decay fungi consisted of the genera *Gymnopilus, Gymnopus, Hypholoma*, and *Pluteus.* Saprotrophic litter decay fungi consisted of the genera *Agaricus, Clitocybe, Hygrocybe, Mycena, Ramariopsis*, and *Rhodocollybia* (see Supplementary Material for species information). Due to low collection numbers in 2012, data from fertilized and unfertilized plots were included in our analysis of the effects of elevated CO_2_, and fertilization and the interactive effect of CO_2_ and fertilization were investigated as well. Samples collected from years before CO_2_ fumigation shutoff were also pooled due to low collection numbers in 2000, 2001, 2002, and 2010.

### Sporocarp Compound Extractions

Chemical extractions were done for a subset of the sporocarps and focused on genera collected in 2012 that were most abundant, and for which we had multiple replicates per genus, specifically *Amanita, Chroogomphus, Lactarius, Russula, Cortinarius, Hydnellum, Suillus, Tylopilus, Gymnopus, Ramariopsis*, and *Rhodocollybia*. Approximately 200 mg of sporocarp material was used to extract protein and structural material using a revised extraction method (Hobbie et al., 2013a). Ground sample was placed in borosilicate culture tubes with 3 mL of *n*-hexanes, capped and mixed. Tubes were incubated at 80°C for 30 min and then centrifuged at 2000 rpm for 5 min. Supernatant was discarded and the wash was repeated two or more times with *n*-hexanes. When the *n*-hexanes solvent was clear after mixing with the sample, final *n*-hexanes were discarded to remove all non-polar compounds and samples were dried down in a Genevac EZ-2.2 Series vacuum evaporator (Genevac Inc., New York, USA) at 45°C. Subsequently, soluble polar compounds were removed by washing samples three or more times with 3 mL of 80% ethanol, using the same method as *n*-hexanes, until the solvent was clear when mixed with samples. After samples were dried down, proteins were hydrolyzed by adding 2 mL of 6 M HCl to the *n*-hexane-washed and ethanol-washed samples, agitated until mixed and incubated at 110°C for 24 h. After hydrolysis, samples were dried down in the vacuum evaporator. Dried samples were then resuspended in 2 mL of 0.01 M HCl and sonicated for 30 min. Tubes were then centrifuged at 2000 rpm for 5 min.

To separate protein and structural compounds the above extracts were washed through cation-exchange columns. Columns were first prepared by washing Dowex 50 WX8 200-400cl-mesh ion exchange resin with 80% ethanol, followed by 6 M HCl and then water until pH was restored to 6. Washed ion exchange resin was placed into sterile 5 mL glass volumetric pipettes to the 4 mL line of the pipette. Columns were rinsed again two times with water, followed by the addition of the supernatant of the resuspended sample extract (0.01 M HCl) to the top of the column. Samples were first eluted with 24 mL of water to remove negatively charged and uncharged molecules. Hydrolyzed proteins were then eluted with 24 mL of 6 M HCl, collected and dried down in the vacuum evaporator. The remaining structural material pellet in the original sample tube was incubated with 2 mL of 2 M NaOH at 110°C for 2 h. The tube was then centrifuged and the supernatant was discarded. The structural material was washed with water several times to remove NaOH and then dried in the vacuum evaporator.

### Isotope Analysis

Ground soil, sporocarps, protein, and structural extracts were analyzed for %C, %N, δ^13^C, and δ^15^N using a Costech 4010 Elemental Analyzer coupled to a Thermo Delta Plus XP IRMS at the University of New Hampshire. Standard deviations of laboratory standards (tuna, Underhill Oa, Underhill Bs, NIST 1515 apple leaves, and NIST 1575a pine needles) for δ^15^N and δ^13^C averaged less than 0.2‰.

### Statistical Analysis

Data were analyzed using JMP Pro 11 software (SAS, Cary, NC, USA). Fungal samples were treated as individual samples, without averaging by ring. Effects of CO_2_ treatment on δ^15^N and δ^13^C of soils and fungi as well as differences between fungal types were explored using linear regression models. Factors of interest included CO_2_ fumigation, fertilization, and an interaction of the two factors as well as functional types for fungal data. Fertilization was also included to test whether samples from fertilized and unfertilized plots should be pooled. Experimental ring was included as a random effect. To identify factors that controlled differences in δ^13^C between protein and structural material, we used a stepwise linear regression model including CO_2_ fumigation, fertilization, bulk fungal δ^15^N and δ^13^C and difference in protein and structural material δ^15^N, %N, and %C.

## Results

### Soil δ^13^C and δ^15^N Analysis

**FIGURE 2:**
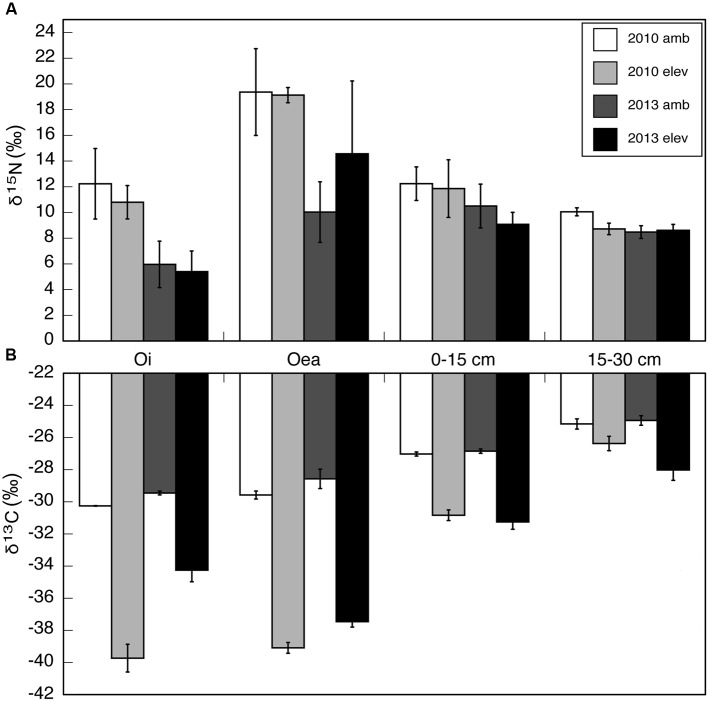
**(A)** δ^15^N and **(B)** δ^13^C of soils collected in 2010 during CO_2_ fumigation and in 2013 three years after CO_2_ fumigation shutoff in both ambient and CO_2_-fumigated plots. Soils were separated at the Oi and Oea organic horizons and at 0–15 and 15–30 cm depths. Each bar represents an average of four replicates in 2010 and three replicates in 2013. Error bars indicate SE.

Soils among all collected horizons were lower in δ^13^C in CO_2_-fumigated plots than in ambient plots both in 2010 during CO_2_ fumigation by a mean of 6.7‰ and in 2013 by 5.2‰. CO_2_ fumigation significantly lowered soil δ^13^C (*p* < 0.0001, *n* = 84) while fertilization and the interaction between fumigation and fertilization did not significantly affect soil δ^13^C values. Depth also affected soil δ^13^C (*p* < 0.0001, *n* = 84) and response to CO_2_ fumigation, with δ^13^C of the Oi horizon in CO_2_-fumigated plots 5.5‰ lower in 2010 than in 2013 (*p* = 0.0150, *n* = 10) (**Figure 2**). In the Oea horizon, δ^13^C of soils in CO_2_-fumigated plots was 1.6‰ lower in 2010 than in 2013 (*p* = 0.0213, *n* = 10). In contrast, the δ^13^C of both mineral soil horizons (0-15 cm and 15-30 cm) did not change between 2010 and 2013. Fertilization and the interaction of fertilization with fumigation were not significant factors (*p* = 0.9232 and 0.8109, respectively). In 2010, the Oi horizon was the most ^13^C-depleted horizon in CO_2_-fumigated plots, while in 2013 the Oea horizon was the most ^13^C-depleted horizon.

To clarify sources of fungal carbon and determine whether carbon from soil organic (C:N bonded) matter was acquired from the younger Oi or older Oea horizons, soil δ^15^N was also investigated. CO_2_ treatment and fertilization did not significantly affect soil δ^15^N, but depth of soil was a significant factor (0.0058, *n* = 84). The Oea horizon was enriched in ^15^N relative to the Oi horizon by 6.4‰ in 2010 and by 5.3‰ in 2013 (**Figure 2**).

### Bulk δ^13^C Analysis

During CO_2_ fumigation in 2010, fungal functional type and fumigation significantly affected bulk δ^13^C (*p* < 0.0001 for both, *n* = 245). All fungal types had lower δ^13^C in CO_2_-fumigated plots than in ambient plots, with the largest difference in sporocarps of fungi with hydrophilic ectomycorrhizae (-11.4‰), followed by hydrophobic ectomyorrhizae (-10.6‰), saprotrophic litter decay fungi (-9.0‰) and wood decay (-4.1‰) fungi (**Table 1**). In CO_2_-fumigated plots, δ^13^C was 3.1 to 8.1‰ higher for fungi with hydrophobic ectomycorrhizae and 4.5 to 9.5‰ higher for fungi with hydrophilic ectomycorrhizae than for litter decay fungi and wood decay fungi. In 2012, 2 years after CO_2_ fumigation shutoff, fungal type and fumigation continued to significantly affect bulk δ^13^C (*p* = 0.0004 and <0.0001 respectively, *n* = 86). Litter decay and wood decay fungi from CO_2_-fumigated plots reflected ^13^C patterns in the Oi and Oea soil horizons and were still 5.8 and 8.6‰ lower in δ^13^C than sporocarps from ambient plots, respectively (**Table 1**). Two years after CO_2_ fumigation shutoff, ectomycorrhizal sporocarps had increased by 10 to 11‰ in ^13^C in formerly CO_2_-fumigated plots. Fertilization and the interaction with fumigation did not significantly affect bulk δ^13^C before or after CO_2_ fumigation shutoff.

**Table 1:** δ^13^C for bulk sporocarps of fungi with hydrophilic ectomycorrhizae (Hi), hydrophobic ectomycorrhizae (Ho), saprotrophic litter decay (ld), and wood decay (wd) collected in ambient and CO_2_-fumigated plots at the Duke Forest FACE site during CO_2_ fumigation in 2010 and 2 years after CO_2_ fumigation shutoff.

	During CO_2_ fumigation			After CO_2_ shutoff		
	δ^13^C_Ambient_ (_‰_)	δ^13^C_Elevated_ (_‰_)	*n*	δ^13^C_Ambient_ (_‰_)	δ^13^C_Elevated_ (_‰_)	*n*
Hi	-27.2 ± 0.4^a^	-38.6 ± 0.3^b^	106	-27.0 ± 0.2^a^	-27.1 ± 0.2^a^	25
Ho	-26.6 ± 0.6^a^	-37.2 ± 0.4^b^	53	-26.5 ± 0.2^a^	-27.0 ± 0.3^a^	11
ld	-25.1 ± 0.6^a^	-34.1 ± 0.6^b^	51	-25.2 ± 0.9^a^	-31.0 ± 0.9^b^	23
wd	-25.0 ± 0.5^a^	-29.1 ± 0.6^b^	29	-25.7 ± 1.1^a^	-34.3 ± 1.0^b^	26

*Significant differences in values due to FACE treatment effects during CO_2_ fumigation and after CO_2_ fumigation shutoff are shown for P < 0.1*.

### Protein and Structural Sporocarp δ^13^C Analysis

**FIGURE 3:**
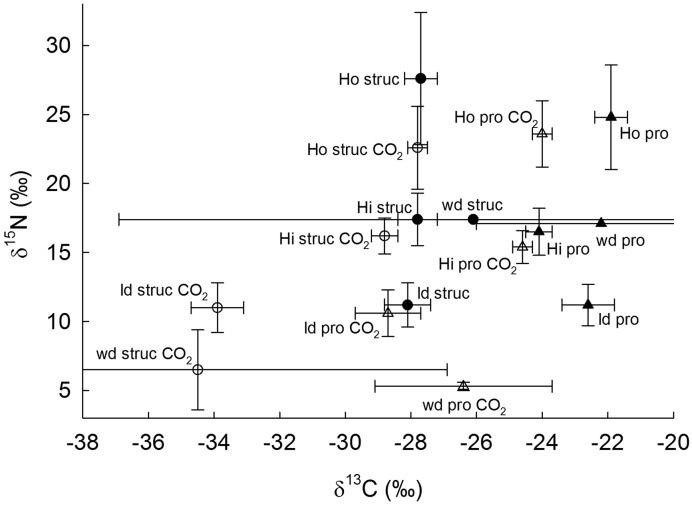
**δ^13^C and δ^15^N of sporocarps of fungi with hydrophobic ectomycorrhizae (Ho) and hydrophilic ectomycorrhizae (Hi) as well as saprotrophic wood decay (wd) and litter decay (ld) fungi collected in ambient (filled symbols) and CO_2_-fumigated plots (CO_2_, open symbols) at the Duke Forest FACE site 2 years after CO_2_ fumigation shutoff.** Error bars indicate SE.

We analyzed protein and structural material of sporocarps collected 2 years after CO_2_ shutoff. CO_2_ fumigation and fungal functional type significantly affected protein δ^13^C (*p* = 0.0253 and 0.0153 respectively, *n* = 37), while fertilization and the interaction of CO_2_ fumigation with fertilization were not significant factors. Protein δ^13^C of sporocarps with hydrophobic ectomycorrhizae was 2.1‰ lower in CO_2_-fumigated plots than in ambient plots (**Table 2**; **Figure 3**). Protein of litter fungi was also lower in δ^13^C in CO_2_-fumigated plots by 6.1‰. Resolution of protein and structural material from wood decay fungi was poor and standard error was high due to a low number of samples (*n* = 3). Only fungal functional type significantly affected δ^13^C of structural material and structural material only differed in δ^13^C between ambient and CO_2_-fumigated plots for litter decay fungi (**Table 2**; **Figure 3**). In addition, in ambient plots, protein of fungi with hydrophilic ectomycorrhizae was lower in δ^13^C than protein of fungi with hydrophobic ectomycorrhizae, litter fungi, and wood decay fungi by 2.4, 1.5, and 1.9‰, respectively.

**Table 2:** δ^13^C for protein and structural material of sporocarps and the δ^13^C difference between the two (δ^13^C_pro-struc_) for fungi with hydrophilic ectomycorrhizae (Hi), hydrophobic ectomycorrhizae (Ho), and saprotrophic sporocarps, specifically litter decay (ld) and wood decay (wd) fungi, collected in ambient and CO_2_-fumigated plots at the Duke Forest FACE site 2 years after CO_2_ fumigation shutoff.

After CO_2_ shutoff	Protein material		Structural material		Protein/Structural		
	δ^13^C_Ambient_ (_‰_)	δ^13^C_Elevated_ (_‰_)	δ^13^C_Ambient_ (_‰_)	δ^13^C_Elevated_ (_‰_)	δ^13^C_pro-strucAmbient_ (_‰_)	δ^13^C_pro-strucElevated_ (_‰_)	*n*
Hi	-24.1 ± 0.4^a^	-24.6 ± 0.3^a^	-27.8 ± 0.6^a^	-28.8 ± 0.4^a^	3.6 ± 0.6^a^	4.2 ± 0.4^a^	20
Ho	-21.9 ± 0.5^a^	-24.0 ± 0.3^b^	-27.7 ± 0.5^a^	-27.8 ± 0.3^a^	5.7 ± 0.8^a^	3.8 ± 0.5^b^	7
ld	-22.6 ± 0.8^a^	-28.7 ± 1.0^b^	-28.1 ± 0.7^a^	-33.9 ± 0.8^b^	5.5 ± 0.6^a^	5.2 ± 0.7^a^	7
wd	-22.2^a^	-26.4 ± 2.7^a^	-26.1^a^	-34.5 ± 7.6^a^	4.0^a^	8.1 ± 4.6^a^	3

*Significant differences in values due to FACE treatment effects are shown for P < 0.05 for protein and structural material and P < 0.1 for protein/structural material.*

The ^13^C enrichment of protein relative to structural material (δ^13^C_pro-struc_) of fungi with hydrophobic ectomycorrhizae was 3.8‰ in CO_2_-fumigated plots and 5.7‰ in ambient plots (**Table 2**). Controls over δ^13^C_pro-struc_ across treatment and fungal type were investigated using stepwise multiple regressions (**Table 3**). Bulk sporocarp δ^13^ and δ^15^N and %N_pro-struc_ were significant factors that accounted for 29.56, 28.30, and 18.39% of the variance, respectively. The adjusted *r*^2^ versus the independent variables was 0.547 for δ^13^C_pro-struc_ (overall *p* = 0.0002).

**Table 3:** Controls over δ^13^C_pro-struc_ (*n* = 27) were investigated using stepwise multiple regressions.

	δ^13^C_pro-struc_ coefficient		
Independent variable	Estimate (‰)	% variance	*p*
Bulk δ^13^C	-0.27 ± 0.10	29.56	*0.0095*
Bulk δ^15^N	-0.16 ± 0.06	28.30	*0.0109*
%N_pro-struc_	-0.30 ± 0.13	18.39	*0.0354*
Functional group (Hi & ld-Ho)	-1.48 ± 0.58	23.76	*0.0183*

*The model explained 0.547 of the variance (p = 0.0002). The % variance is the % of explained variance attributed to the specified independent variable in the regression. P-values are italicized when P < 0.05.*

## Discussion

Identifying sources of carbon is important for distinguishing different functional types of fungi. Stable isotope analysis of bulk fungal tissue has been used to determine that ectomycorrhizal and saprotrophic fungi rely primarily on litter and photosynthates as carbon sources (Hobbie et al., 2001, 2014; Mayor et al., 2009). Isotopic relationships between bulk sporocarps collected in ambient and CO_2_-fumigated plots in a previous Duke FACE collection also suggested that ectomycorrhizal fungi derived carbon from soil and litter (Hobbie et al., 2014). Here, we clarified fungal sources of carbon that are difficult to detect in bulk tissue isotope analysis by combining information from bulk tissue, protein and structural extracts of sporocarps from the Duke FACE experiment.

As predicted, bulk sporocarps from all four functional classes collected during ^13^C-depleted CO_2_ fumigation were low in δ^13^C relative to ambient samples, while only saprotrophic fungi maintained low δ^13^C signatures after CO_2_ shutoff. The persistently low bulk δ^13^C of wood decay fungi and litter decay fungi from CO_2_-fumigated plots 2 years after fumigation shutoff indicated that these fungi relied on surficial litter and wood produced during CO_2_ fumigation. The smaller difference in δ^13^C between pre-fumigation and post-fumigation samples of wood decay fungi than of litter decay fungi also suggested that wood decay fungi may use carbon that was produced before CO_2_ fumigation was initiated, as wood C sources can be relatively old. The increased δ^13^C of fungi with hydrophobic and hydrophilic ectomycorrhizae in CO_2_-fumigated plots 2 years after fumigation shutoff and similar values to fungi in ambient plots indicate that these functional groups relied on recent plant photosynthate that was produced after fumigation as a carbon source. However, these bulk tissue results lack sufficient sensitivity to assess our hypothesis that ectomycorrhizal fungi access carbon in soil-derived organic nitrogen pools, which are present in deeper soil horizons and ^13^C-enriched relative to other carbon pools.

In contrast to bulk analyses, combined carbon and nitrogen isotopic analysis of fungal protein of sporocarps collected 2 years after CO_2_ fumigation shutoff supported our hypothesis that fungi with hydrophobic ectomycorrhizae use soil-derived organic nitrogen sources, such as humic substances, proteinaceous materials, amino sugars and heterocyclic nitrogen compounds, for protein carbon. In ambient plots, protein from both litter decay fungi and fungi with hydrophobic ectomycorrhizae were enriched in ^13^C relative to protein from wood decay fungi and fungi with hydrophilic ectomycorrhizae, suggesting that the former fungi used carbon from relatively older and deeper ^13^C-enriched organic soil pools. The ^13^C-depleted values of protein from fungi with hydrophilic ectomycorrhizae relative to fungi with hydrophobic ectomycorrhizae in ambient plots suggest that carbon from recent plant photosynthate was incorporated into protein of fungi with hydrophilic ectomycorrhizae. Furthermore, in plots previously exposed to elevated CO_2_, protein of litter decay fungi and fungi with hydrophobic ectomycorrhizae was depleted in ^13^C relative to ambient plots, also suggesting that these fungal groups assimilate older organic nitrogen that was synthesized during CO_2_ fumigation years. However, δ^15^N patterns in protein revealed that fungi with hydrophobic ectomycorrhizae use older organic nitrogen than litter decay fungi, since protein of ectomycorrhizal fungi was ^15^N-enriched relative to saprotrophic fungi, resembling the ^15^N-enriched organic nitrogen in the older Oea soil horizon more than the Oi soil horizon. Although ectomycorrhizal fungi are higher in δ^15^N than saprotrophic fungi due to ^15^N enrichment during transfer of ^15^N-depleted nitrogen to plants (Hobbie and Colpaert, 2003), the 5‰ higher δ^15^N of protein of fungi with hydrophobic ectomycorrhizae compared to fungi with hydrophilic ectomycorrhizae also supports our hypothesis that fungi with hydrophobic ectomycorrhizae use older ^15^N -enriched pools of organic nitrogen.

Although exploration of the effect of genus in addition to functional type on fungal isotopic response to CO_2_ fumigation would have provided additional information on the role of different fungi in carbon cycling, this was beyond the scope of our study due to the limited number and variety of sporocarps that generated within experimental plots. Regardless, our results support prior results on radiocarbon of protein of ectomycorrhizal fungal protein indicating that fungi with hydrophobic ectomycorrhizae use older organic nitrogen sources than fungi with hydrophilic ectomycorrhizae (Hobbie et al., 2013a). These isotopic carbon patterns likely arise from differences in enzymatic capacity and preference for insoluble or soluble substrates between hydrophobic and hydrophilic ectomycorrhizae (Lilleskov et al., 2011; Hobbie et al., 2013b). Although the ^13^C enrichment of saprotrophic wood and litter decay protein relative to hydrophilic protein in ambient plots suggests use of organic nitrogen by saprotrophic fungi, these patterns could also be derived from litter or wood cellulose and *de novo* synthesis of fungal amino acids. Therefore, further investigation is needed of carbon sources for protein and structural material of saprotrophic fungi, such as with separate ^13^C labeling of plant cellulose and organic nitrogen, in order to determine the effect of fungal preference for different carbon substrates as well as age of carbon substrates on fungal isotopic patterns.

The δ^13^C of structural material of fungi with hydrophobic and hydrophilic ectomycorrhizae suggests that ectomycorrhizal fungi rely solely on recent plant photosynthates for structural carbon, since the low δ^13^C from CO_2_ fumigation did not persist 2 years after CO_2_ shutoff. Additionally, the δ^13^C of structural material of saprotrophic fungi was 5‰ lower than ectomycorrhizal fungi, since litter decay fungi rely heavily on surficial litter that persisted after CO_2_ shutoff as a carbon source.

The δ^13^C_pro-struc_ ranged from 3.6 to 5.7‰, overlapping the estimated range of ^13^C enrichment between protein and structural carbohydrates of 4.2 ± 0.5‰ in Hobbie et al. (2012). Similar to this previous study on protein and carbohydrate content in caps and stipes of sporocarps, nitrogen content and δ^15^N between protein and structural material correlated with δ^13^C_pro-struc_ in the current study. We previously hypothesized that ^13^C differences between fungal protein and carbohydrates could primarily be due to differences in carbon metabolism (Hobbie et al., 2012). Specifically, ^13^C fractionation and loss of ^13^C-depleted CO_2_ was hypothesized during production of carbon skeletons from the tricarboxylic acid cycle for protein synthesis (O’Leary and Yapp, 1978; Grissom and Cleland, 1988; Tcherkez et al., 2003). However, correlation of nitrogen content and δ^15^N with δ^13^C of protein and structural material in our data suggests that nitrogen source is also an important factor. Differences in δ^13^C_pro-struc_ between ambient and previously CO_2_-fumigated plots for fungi with hydrophobic ectomycorrhizae also suggest that these materials have different carbon sources. If ^13^C enrichment of protein were due to internal fractionation rather than differences in C source, δ^13^C_pro-struc_ should be the same for ambient and CO_2_-fumigated plots. Although δ^13^C_pro-struc_ was similar for ambient and CO_2_-fumigated plots in saprotrophic fungi and fungi with hydrophilic ectomycorrhizae, likely due to the use of more recently produced carbon sources, δ^13^C_pro-struc_ of fungi with hydrophobic ectomycorrhizae was lower by 1.9‰ in plots that were previously fumigated with elevated CO_2_. This difference between protein and structural material of hydrophobic ectomycorrhizal fungi in ambient and CO_2_ fumigation treatments indicates that, in CO_2_-fumigated plots, the ^13^C-depleted carbon in protein was derived from soil organic nitrogen that was synthesized during CO_2_ fumigation. Our results therefore indicate that fungi with hydrophobic ectomycorrhizae use both organic nitrogen and recent photosynthate as a carbon source. However, this should be investigated further with more fungi with hydrophobic and hydrophilic ectomycorrhizae.

Movement of recently fixed carbon is rapid through plants and the sporocarp δ^13^C of ectomycorrhizal fungi returned to natural levels as quickly as 2-3 months after pulse ^13^C labeling of host plants (Högberg et al., 2010). Isotopic differences in CO_2_-fumigated plots between ectomycorrhizal and saprotrophic fungi during and after fumigation therefore probably appeared shortly after fumigation shutoff. This difference could be used to identify compounds derived from recent photosynthate. However, the 2-year period after CO_2_ fumigation shutoff in this study also allowed us to identify which functional groups were assimilating newly produced photosynthate, young organic nitrogen in surficial litter or old organic nitrogen sources in deeper soils. Future studies in global change experiments of δ^13^C values of bulk tissue, protein and structural material before CO_2_ fumigation, shortly after fumigation shutoff and a few years after fumigation shutoff can accordingly further help to clarify fungal sources of carbon.

## Conclusion

Our data demonstrate that protein and structural material can use different carbon pools and that patterns of carbon use differ among fungi with hydrophilic ectomycorrhizae, fungi with hydrophobic ectomycorrhizae, and saprotrophic fungi. Specifically, fungi with hydrophilic ectomycorrhizae used recent plant photosynthate and young organic matter for carbon while fungi with hydrophobic ectomycorrhizae mined old organic material for protein carbon and converted recent plant photosynthate into structural material. These results demonstrate that prior inferences about differences in organic nitrogen partitioning of fungi with hydrophobic and hydrophilic ectomycorrhizae reported from boreal forests (Hobbie et al., 2013a) apply to a much warmer temperate forest ecosystem as well. Our data also demonstrate for the first time that litter decay fungi used young organic matter carbon for both protein and structural material.

## Author Contributions

JC is the primary author and conducted the laboratory and data analysis of this study. KH provided samples and the experimental site. EH provided samples and the initial concept for this study. All authors contributed to the composition of this manuscript.

## Conflict of Interest Statement

The authors declare that the research was conducted in the absence of any commercial or financial relationships that could be construed as a potential conflict of interest.
